# Supplementary figures and images for: Rv3839-Rv3840 links the endogenous heme biosynthesis pathway with Mycobacterium tuberculosis adaptation to nitric oxide and iron limitation stress
Source: PLoS Genet. 2026 Jun 8;22(6):e1012202. doi: 10.1371/journal.pgen.1012202 (PMC13258150; doi:10.1371/journal.pgen.1012202)

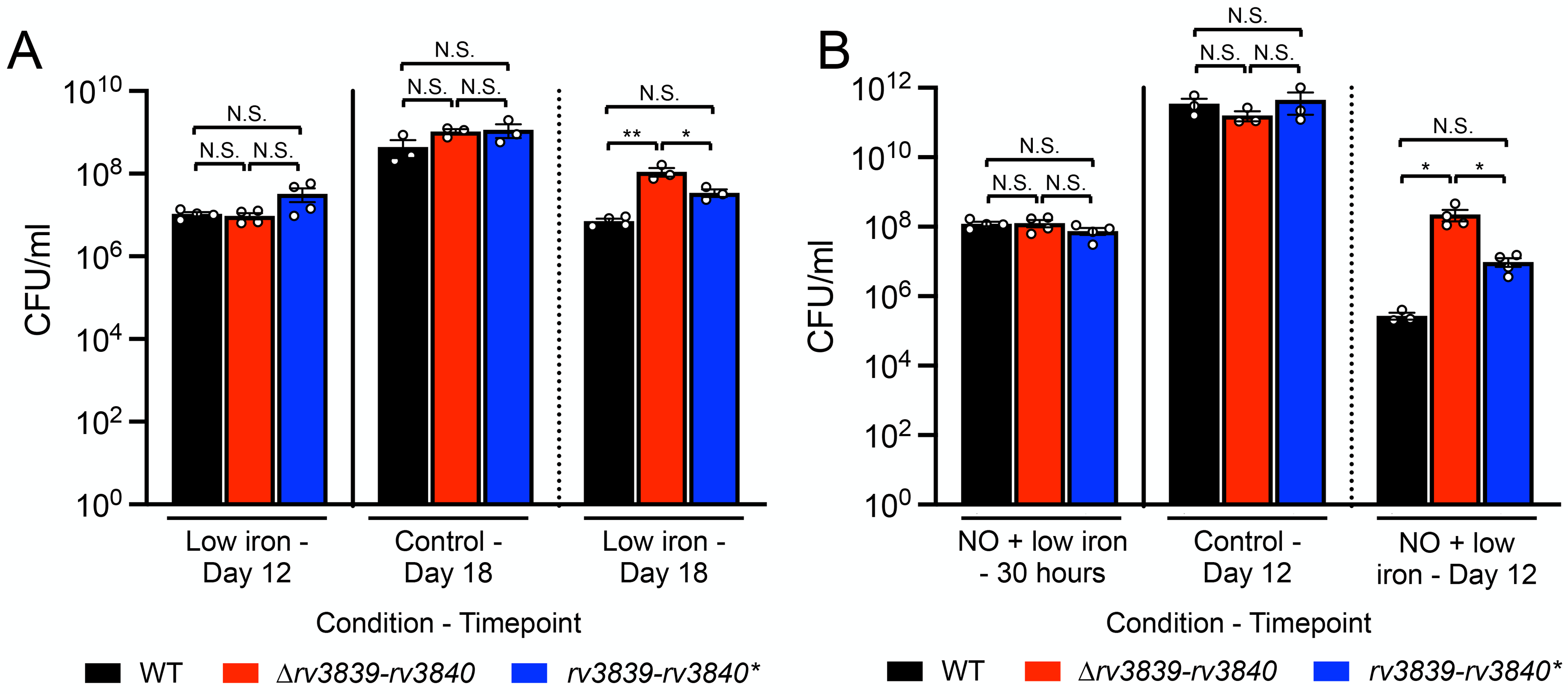

Supplement: S1 Fig — (A) Growth of Δrv3839-rv3840 Mtb is less restricted under iron limitation than WT Mtb. WT, Δrv3839-rv3840, and rv3839-rv3840* (complemented strain) Mtb were cultured in 7H9, pH 7.0 media (control) or iron-depleted minimal media with 100 µM 2’2’-dipyridyl (low iron) for 12 days. At day 12, the strains were sub-cultured at OD600 = 0.05 into the same medium. Aliquots from the low iron condition were plated for CFUs at day 12 (after the sub-culture to the same starting OD600 = 0.05 for all strains) or 18, and from the control condition at day 18. Data are shown as means ± SEM from 3-4 experiments. (B) Δrv3839-rv3840 Mtb prematurely exits NO and low iron stress-induced growth arrest. WT, Δrv3839-rv3840, and rv3839-rv3840* Mtb were grown in aerated conditions in 7H9, pH 7.0 and sub-cultured in either 7H9, pH 7.0 (control) or in iron-depleted minimal media with 100 µM 2’2’-dipyridyl and treated with 6 doses of 100 µM DETA NONOate over 30 hours (NO + low iron). Aliquots from the NO + low iron condition were plated for CFUs at 30 hours (after the last dose of DETA NONOate) or day 12, and from the control condition at day 12. Data are shown as means ± SEM from 3-4 experiments. p-values in both (A) and (B) were obtained with a one-way ANOVA with Tukey’s multiple comparisons. N.S. not significant, * p < 0.05, ** p < 0.01. The numerical data underlying the graphs shown in this figure are provided in S1 Data. (TIF) [file pgen.1012202.s001.tif]

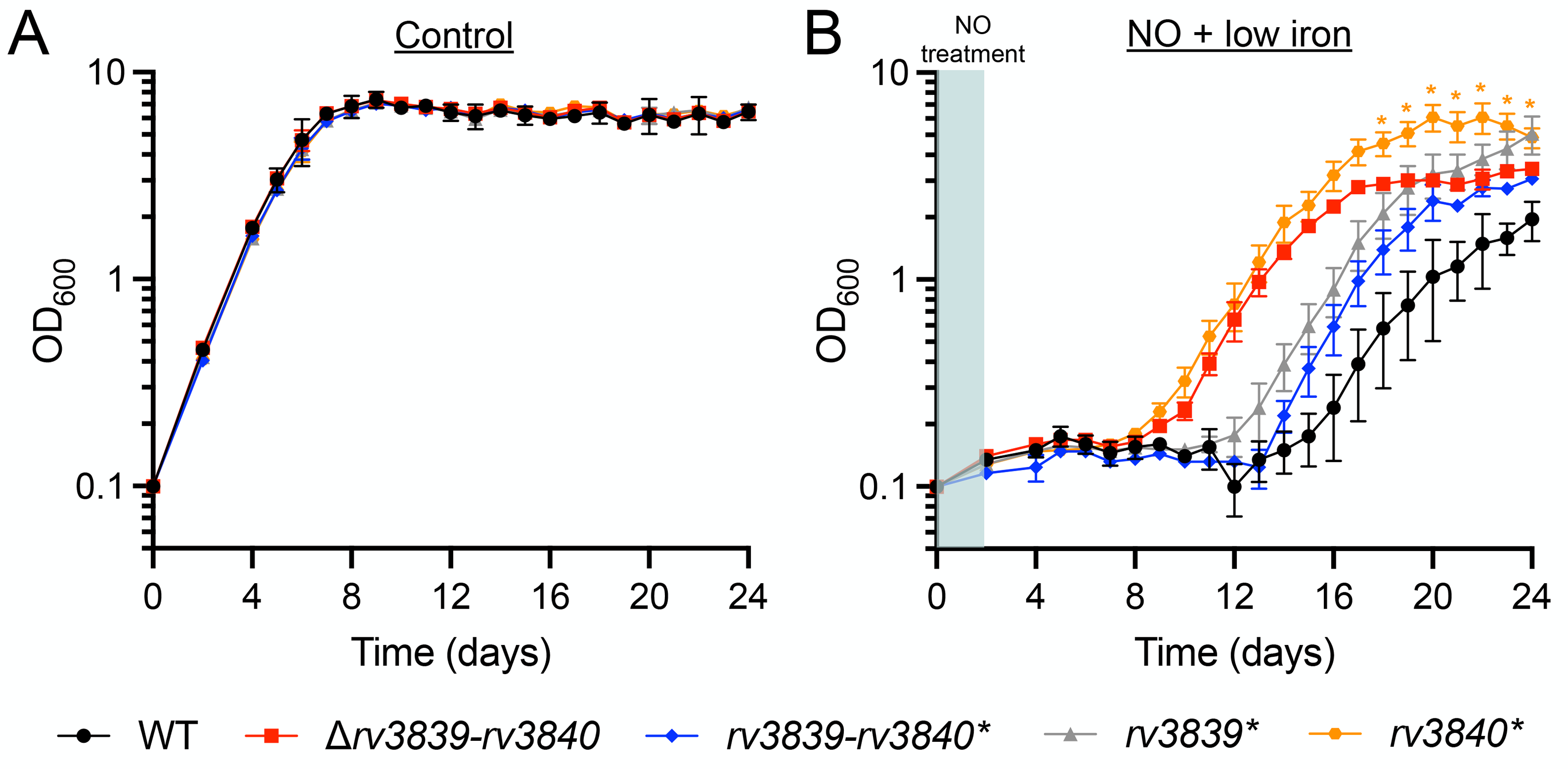

Supplement: S2 Fig — WT, Δrv3839-rv3840, rv3839-rv3840*, rv3839*, and rv3840* Mtb were grown in aerated conditions in 7H9, pH 7.0 and sub-cultured in either 7H9, pH 7.0 (A, control), or in iron-depleted minimal media with 100 µM 2’2’-dipyridyl and treated with 6 doses of 100 µM DETA NONOate (B, NO + low iron) over 30 hours (shaded area). Bacterial growth was tracked by OD600 every day for 24 days. Data are shown as means ± SEM from 4-8 experiments. p-values in (B) were obtained with unpaired t-tests with Welch’s correction, comparing rv3840* to ∆rv3839-rv3840 Mtb. All comparisons of rv3839* to rv3839-rv3840* Mtb were non-significant. * p < 0.05. The numerical data underlying the graphs shown in this figure are provided in S1 Data. (TIF) [file pgen.1012202.s002.tif]
